# Classification of X-Ray Attenuation Properties of Additive Manufacturing and 3D Printing Materials Using Computed Tomography From 70 to 140 kVp

**DOI:** 10.3389/fbioe.2021.763960

**Published:** 2021-11-29

**Authors:** Xiangjie Ma, Martin Buschmann, Ewald Unger, Peter Homolka

**Affiliations:** ^1^ Center for Medical Physics and Biomedical Engineering, Medical University of Vienna, Vienna, Austria; ^2^ Division of Medical Radiation Physics, Department of Radiation Oncology, Medical University of Vienna, Vienna, Austria

**Keywords:** additive manufacturing, 3D printing, computed tomography, quality control, radiographic phantoms, x-ray, phantom materials

## Abstract

Additive manufacturing and 3D printing is particularly useful in the production of phantoms for medical imaging applications including determination and optimization of (diagnostic) image quality and dosimetry. Additive manufacturing allows the leap from simple slab and stylized to (pseudo)-anthropomorphic phantoms. This necessitates the use of materials with x-ray attenuation as close as possible to that of the tissues or organs mimicked. X-ray attenuation properties including their energy dependence were determined for 35 printing materials comprising photocured resins and thermoplastic polymers. Prior to measuring x-ray attenuation in CT from 70 to 140 kVp, printing parameters were thoroughly optimized to ensure maximum density avoiding too low attenuation due to microscopic or macroscopic voids. These optimized parameters are made available. CT scanning was performed in a water filled phantom to guarantee defined scan conditions and accurate HU value determination. The spectrum of HU values covered by polymers printed using fused deposition modeling reached from −258 to +1,063 at 120 kVp (−197 to +1,804 at 70 kVp, to −266 to +985 at 140 kVp, respectively). Photocured resins covered 43 to 175 HU at 120 kVp (16–156 at 70, and 57–178 at 140 kVp). At 120 kVp, ASA mimics water almost perfectly (+2 HU). HIPS (−40 HU) is found close to adipose tissue. In all photocurable resins, and 17 printing filaments HU values decreased with increasing beam hardness contrary to soft tissues except adipose tissue making it difficult to mimic water or average soft tissue in phantoms correctly over a range of energies with one single printing material. Filled filaments provided both, the HU range, and an appropriate energy dependence mimicking bone tissues. A filled material with almost constant HU values was identified potentially allowing mimicking soft tissues by reducing density using controlled under-filling. The measurements performed in this study can be used to design phantoms with a wide range of x-ray contrasts, and energy dependence of these contrasts by combining appropriate materials. Data provided on the energy dependence can also be used to correct contrast or contrast to noise ratios from phantom measurements to real tissue contrasts or CNRs.

## Introduction

Due to their great potential, additive manufacturing and 3D printing have become indispensable technologies in medicine as a whole, in medical imaging in particular. This is not only due to the increasing number of developers and scientists eagerly embracing the new method for realizing their projects, but also by the rapid technological development of 3D printing processes and materials available to the imaging community. Based on the number of publications abstracted in the Web of Science (Clarivate Analytics LLC, Philadelphia, United States) in the last 3 years (2019 to August 2021), Radiology, Nuclear Medicine and Medical Imaging ranked just behind Multidisciplinary Material Science (#1), Biomedical Engineering (#2), Biomaterials (#3), Surgery (#4), and Dentistry (#5), when purely technical fields just applying medical imaging and related methods were omitted. These omissions include applied physics and engineering manufacturing research, where additive manufacturing and medical/dental appliances were used. The increasing importance of additive manufacturing and 3D printing in medical and dental applications as a whole, and in medical imaging in particular, is demonstrated best not only by the total number of indexed scientific publications, but also by their relative share, where a linear rise can be seen in the last decade. For both, 3D printing and additive manufacturing in medical and dental applications, and in medical imaging, the relative share of all indexed scientific publications increased by over 40% in 3 years (from 2017 to 2020).

In medical imaging, the most important fields where 3D printing and additive manufacturing are applied include design and production of phantoms for all kinds of modalities including CT, US, nuclear medicine modalities like PET and SPECT, projective imaging and mammography (Filippou and Tsoumpas, 2018). In 2D and 3D X-Ray imaging and dosimetry, 3D printing and additive manufacturing paved the way from rather simple phantoms, like stylized phantoms (Smet et al., 2018) to almost anthropomorphic phantoms for projective imaging (Irnstorfer et al., 2019), mammography (Carton et al., 2011; Kiarashi et al., 2015; Schopphoven et al., 2019) or dosimetry (Homolka et al., 2017). While simple test objects and phantoms are only capable of assessing technical image quality, advanced phantoms allow progressing towards determination and optimization of task specific diagnostic image quality. Anthropomorphic three dimensional phantoms capable of producing (nearly) realistic tissue background patterns–called “anatomic noise”– in which lesions can be embedded represent a milestone in performing investigations of lesion detectability, optimizing procedure settings and system evaluation (Ivanov et al., 2018). 3D printing has the potential to bring medical physicists developing these phantoms closer to being able to produce these structured anthropomorphic backgrounds (Solomon et al., 2014). On the other hand, printing realistic anthropomorphic lesions with appropriate materials and extremely fine spatial resolutions making them suitable even for mammography and tomosynthesis applications has been shown to be feasible, even if the anatomic background is provided by a “semi-anthropomorphic” model composed of acrylic spheres of various sizes (Cockmartin et al., 2017), or PVC film submerged in a paraffin gel with a non-uniform distribution (Sousa et al., 2018).

However, in medical imaging applications, printing materials are usually selected based on their properties used by the imaging modality to create image signals and contrasts, rather than the properties for which they are typically designed for. Development of these materials is mostly driven by industrial additive manufacturing applications. In medical imaging applications, however, totally different properties are important. These include proton density and relaxation time constants in MRI, acoustic properties and echogenicity in ultrasound imaging, and x-ray attenuation properties in CT and projective x-ray imaging.

Still, and sometimes against the claims in publications, most existing “anthropomorphic phantoms” should rather be viewed as being “semi-” or “pseudo-anthropomorphic”. In order to be really anthropomorphic, a phantom would not only represent the anatomy with the spatial resolution of the phantom at least resembling the intrinsic spatial resolution of the imaging modality, but also would the materials used to represent tissues need to imitate the interaction properties over the full range of acquisition settings of the modality or modalities they are intended for. Limiting the scope to x-ray imaging (projective imaging like general radiography, fluoroscopy and interventional radiology, and computed tomography), this would necessitate that x-ray attenuation was very closely equal to the attenuation of the tissue to be imitated over the full range of X-ray photon energies present in the polychromatic beam, from the minimum to maximum kVp settings used. However, this requirement is usually relaxed by demanding that the total attenuation and the energy dependence of the attenuation shall resemble the energy dependence of the materials or tissues mimicked as closely as possible. However, this is more easily achieved for projective imaging, where in most cases it is sufficient to imitate the mass attenuation coefficient and possibly add air gaps as was done in the simple slab phantoms used for dosimetry and dose audits, like the NEXT and CDRH phantoms (Suleiman et al., 1999; Spelic et al., 2004; IAEA, 2007), than for computed tomography where the linear attenuation coefficient needs to imitated (Homolka and Nowotny, 2002). The latter usually results in the necessity of lowering the mass density if soft tissues or water are imitated by adding filling materials with very low density if curable liquid resins were used (White et al., 1977), or using low density thermoplastic polymers or a mixture of these (Kalender et al., 1988; Homolka and Nowotny, 2002). In castable resin based materials mimicking soft tissue or water, typically hollow air filled phenolic microspheres were used (White et al., 1977).

In the underlaying physics the energy dependence of the x-ray mass attenuation coefficient being equal to the tissue that is mimicked, translates to the ratio of the contribution of inelastic scattering to the photoelectric effect (absorption) being as similar as possible. In contrast to therapeutic photon energies this is in the lower keV range especially complicated by the contribution of the photoelectric effect. However, this can usually be only satisfied for a limited photon energy range, that must include all kVp and filtration settings possibly used. In mammography this includes also all anode filter combinations. Using thermoplastic and resin-based polymers, imitating the x-ray attenuation of soft tissues is especially difficult for photon energies below 30 keV due to the low effective atomic number of the polymers (Homolka et al., 2002). Therefore, phantom materials need to be formulated (if possible) or selected (in case of 3D printing) carefully for the application and photon energy range used. Materials simulating a given tissue with respect to x-ray attenuation at general radiography photon energies will most likely not be suitable at mammography energies, and vice versa. In this work the main focus is characterizing available 3D printing materials with regard to their x-ray attenuation for tungsten spectra from 70 to 150 kVp covering typical photon energies used in general radiography and computed tomography. It needs to be stressed, that in phantoms used for optimizing procedure parameters like beam hardness (kVp and/or filtration, e.g.) it is imperative that the energy dependence of the phantom–and thus, the energy dependence of any important radiographic contrast, SNR or CNR determined–is correct. The same holds true for phantoms used in dosimetry or dose determination, especially if energy dependent dosemeters like TLDs or semiconductor dosemeters are employed, or wide polychromatic spectra are used.

Characterization and also reproducibility of x-ray attenuation properties of additive manufacturing materials is complicated by various issues. Exact chemical composition is often not available to the end user, and it may change in different batches since materials are usually developed further to improve printing results or simplify the printing process. Another issue is found with materials, where printing parameters influence the final mass density of the printout. This needs to be addressed properly, otherwise a too low print density due to a suboptimal parameter setting would be incorrectly attributed to the material and not to the process parameters. This is particularly important for printing processes in which the polymers are melted in air during the printing process, such as FDM (Fused Deposition Modeling) or SLS (Selective Laser Sintering).

The objective of this work was to find and describe optimum printing parameters for a wide range of additive manufacturing materials, and to quantify their x-ray attenuation properties as exactly as possible using CT scans from the lowest to the highest kVp value currently available in our institution.

## Materials and Methods

### Printing Materials and Technologies Used

Thermoplastic polymers available for fused deposition modeling (FDM) printers were printed on an Ultimaker 2+ with standard 0.4 mm nozzle diameter (Ultimaker BV, Utrecht, Netherlands). A wide range of polymer filaments was selected with the scope to include a representative cross section of unfilled base polymers. Filled polymers include filaments with potentially useful x-ray attenuation properties including materials filled with mineral and metal powders potentially allowing mimicking hard tissues in radiographic phantoms.

UV curable resin samples were printed with polyjet and stereolithography (SLA) printers. A Stratasys Connex 3 Objet 500 polyjet printer (Stratasys Ltd., Eden Prairie, United States), a Formlabs Form 2 (Formlabs Inc., Somerville, United States, SLA) and an Anycubic Photon open resin printer (Shenzhen Anycubic Technology Co., Ltd., Shenzhen, China, SLA) were employed. Materials printed with these technologies were chosen to include both, rigid and flexible resins, and represent a cross section of readily available resins from different manufacturers. Ceramic materials were excluded (as also were heavily metal filled FDM filaments, like such filled with metals with higher atomic number than aluminum) since their typically high x-ray attenuation does not allow quantitative measurements in CT due to severe beam hardening and quantum starvation artifacts.

Printing materials and printers used for the respective materials are summarized in Tables 1 (FDM printer) and 2 (Polyjet and SLA printers).

**TABLE 1:** Polymer filaments used on the FDM printer.

		Filament name	Manufacturer	Manufacturer Code/EAN
High attenuation filaments				
Vinyl	Polyvinyl chloride	Vinyl 303 natural	Fillamentum Manufacturing Czech s.r.o., Hulín, Czech Republic	VIN303_285_nat
PLA/stone	PLA filled with 50% powdered stone	StoneFil Pottery Clay	Formfutura BV, Nijmegen, Netherlands	285STONEFIL-PCLAY-050 0
PLA/chalk	PLA with chalk powder	PLA Mineral natural	Fiberlogy SA, Brzezie, Poland	PLA-MIN-NATUR-285-085
Filled filaments with medium and low attenuation				
PLA-PHA/Glow	PLA/PHA filled with phosphorescent pigment	GlowFill	colorFabb BV, Belfeld, Netherlands	8719033555136
PLA-PBAT bio carbon	PLA/PBAT based biocompound with carbon fibers	GreenTEC PRO Carbon	Extrudr FD3D GmbH, Lauterach, Austria	9010241426973
PLA/Al	PLA filled with 10% aluminum powder	Aptofun Metal Filament Aluminium	Aptotec UG, Tübingen, Germany	B01ITNXRWD
PLA/wood	PLA filled with 40% grinded wood particles	EasyWood Birch	Formfutura	285EWOOD-BIRCH-0500
PLA-PHA/Cork	PLA/PHA (Polylactic acid/Polyhydroxyalkanoate) blend filled with cork powder	corkFill	colorFabb	8719033555327
PETG mod./Carbon	HDglass (PETG based polymer blend) with 20% carbon fibers	CarbonFil	Formfutura	175CARBFIL-BLCK-0500
PLA and PLA/PBAT based filaments				
PLA	Polylactic acid	PLA transparent	Ultimaker BV, Utrecht, Netherlands	1614
PLA 2	Polylactic acid	PLA Sparkly Silver	Shenzhen Eryone Technology Co. Ltd., Shenzhen, China	GPLA-SILVER-175-1000
PLA/PBAT bio	PLA/PBAT (polylactic acid/polybutylene adipate terephthalate) based biocompound	GreenTEC PRO natural	Extrudr	9010241426034
PET and PETG based filaments				
PET	Polyethylene terephthalate	EPR InnoPET	Innofil3d BV, Emmen, Netherlands	Pet-0301b075
PET mod.	PET copolyesther, Eastman Amphora 3D Polymer AM1800	XT-Clear	colorFabb	8719033553019
PET mod. 2	PET copolyesther, Eastman Amphora 3D Polymer AM3300	nGen clear	colorFabb	8719033554733
PETG mod.	PETG based polymer blend	HDglass clear	Formfutura	285HDGLA-CLEAR-0750
ABS, ASA and ASA based filaments				
ABS	Acrylonitrile butadiene styrene	ABS transparent	Verbatim	55019
ASA	Acrylonitrile styrene acrylate	ASA Extrafill natural	Fillamentum	00118
ASA mod.	Modified ASA	ApolloX White	Formfutura	285APOX-WHITE-0750
PS, PP, PC and Polyamid based filaments				
HIPS	High impact polystyrene	HIPS wonderous white	ICE Filaments, Ham, Belgium	ICEFIL3HPS170
PP	Polypropylene	PP transparent	Verbatim GmbH, Eschborn, Germany	55951
PP light	Polypropylene with 25% hollow borosilicate glass microspheres	Pegasus PP Ultralight	Formfutura	285PEGAPP-NAT-0500
PC	Polycarbonate	PC-Plus transparent	Polymaker, Shanghai, China	70409
Nylon	Polyamide	Nylon transparent	Ultimaker	1647
PU based filaments				
TPU	Thermoplastic polyurethane	TPU transparent	Extrudr	9010241152001

Notes: Manufacturer details are only shown on first appearance. In case no manufacturer code was available, EAN is stated for identification.

**TABLE 2:** Polyjet and SLA resins used.

	Printer used	Resin name	Manufacturer	Manufacturer code
Rigid SLA resins				
Vero white	Stratasys Objet 500	Vero Pure White	Stratasys Ltd., Eden Prairie, United States	OBJ-03327
Vero clear	Stratasys Objet 500	Vero Clear	Stratasys	OBJ-03271
Vero blue	Stratasys Objet 500	Vero Blue	Stratasys	OBJ-03204
FL clear	Formlabs Form 2	Formlabs Clear Resin	Formlabs Inc., Somerville, United States	RS-F2-GPCL-04
PCre clear	Anycubic Photon	Prima Creator Value UV/DLP resin clear	Prima Printer Nordic AB, Malmö, Sweden	PV-RESIN- B405-CL
AC trans-lucent	Anycubic Photon	Anycubic 3D Printing UV Sensitive Resin Basic, Translucent green	Shenzhen Anycubic Technology Co., Ltd., Shenzhen, China	AB-POT048
Flexible SLA resins				
Tango	Stratasys Objet 500	Tango Plus (translucent)	Stratasys	OBJ-03224
FL flex	Formlabs Form 2	Formlabs Flexible Resin	Formlabs	RS-F2-FLGR-02
FL elastic	Formlabs Form 2	Formlabs Elastic Resin	Formlabs	RS-F2-ELCL-01
PCen flex	Anycubic Photon	PhotoCentric3D UV LCD Resin Flexible clear	Photocentric Ltd., Peterborough, United Kingdom	PHODCL01UVFLEX

The polyjet technology does not allow manual settings of print parameters. The same holds true for SLA samples printed with one of the printers (Formlabs Form 2), since printing materials are recognized by the printer, and parameters set automatically. On the Anycubic Photon SLA printer, print settings recommended by the resin manufacturers were used after verification of their appropriateness from test object printouts.

### Optimization of Printing Parameters in FDM

In FDM general purpose printing, the density of the printed objects is usually below the density of the filament used. Inherent to the printing process, and dependent on the layer thickness and the viscosity of the melted polymer, the deposition of material results in microscopic voids between adjacent polymer deposition lines. In case this effect is strongly present, the layer structure can be seen on the outer surface, and transparent materials appear opaque. However, rough surfaces can also be caused by too strong over-extrusion, so printing parameters are usually optimized for smooth outer surfaces. Unless controlled under-extrusion is used in a reproducible fashion to reduce density and thus x-ray attenuation in radiographic phantoms, it is important to ensure maximum filling ratios, i.e., maximum packing, in the samples used to measure and characterize attenuation properties of printing materials. Optimally, the measured density of the printed samples resembles the filament density.

In this work, starting from printing parameters optimized for general purpose prints, printing parameters were fine tuned to result in the maximum filling ratio, i.e., maximum achievable mass density. Provided the specification of filament density was available and correct, the aim of the parameter optimization was to produce samples with a mass density identical to the filament density. In a first step printing parameters like nozzle temperature and printing speed were optimized using the #3DBenchy test object (#3DBenchy, 2021) available as STL file under the Creative Commons License. The aim of this first step was to achieve optimum printing results including minimal artifacts like stringing, warping, surface roughness, an even surface structure, and dimensional accuracy. In the next step homogeneous cylinders with 15 mm diameter and 20 mm height were printed and their mass density determined gravimetrically. In case density was lower than filament density, over-extrusion was systematically applied by increasing the flow rate. If increasing flow rate up to a point where over-extrusion artifacts became visible or dimensional accuracy decreased did not result in the desired density, layer height was decreased until either the specified mass density was achieved, or until further increase of over extrusion and reduction of layer height did not result in increased density. Layer highs available on the printer used were 0.15, 0.10, and 0.6 mm, respectively.

### Determination of CT Values

**FIGURE 1:**
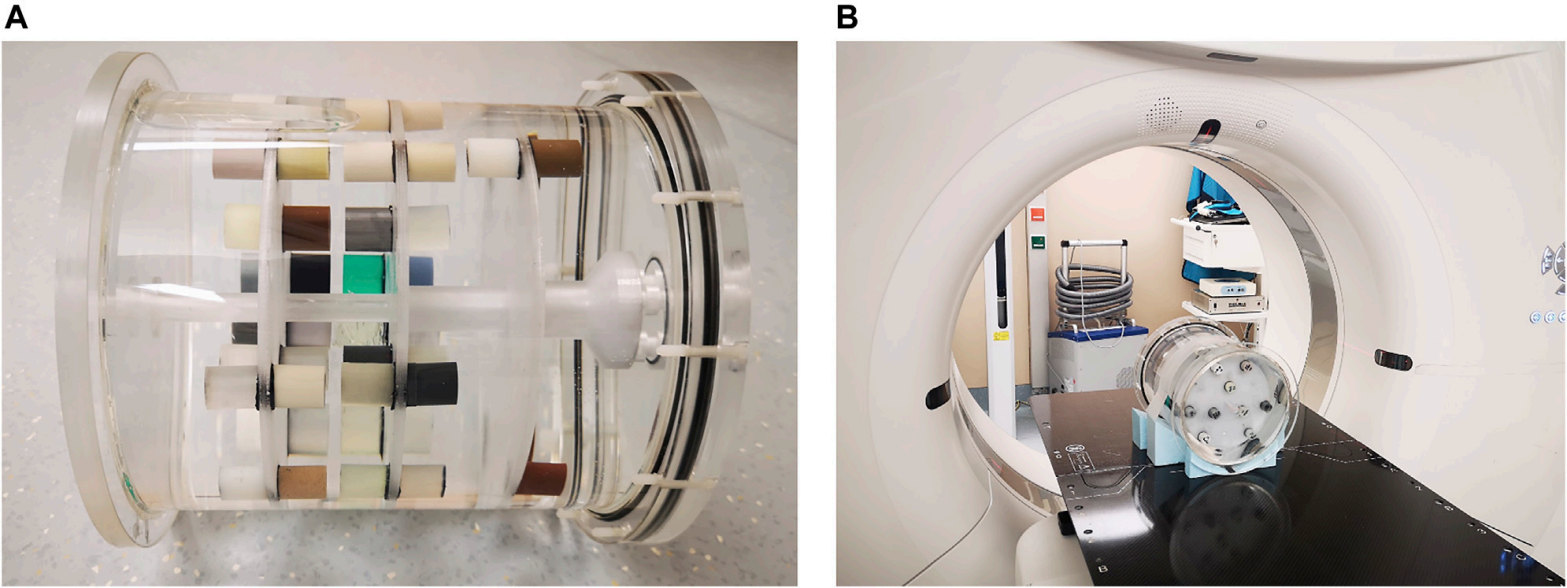
Printed sample cylinders mounted into a water filled phantom for CT scanning.

The printed cylinders were mounted in a water filled phantom for scanning (Figure 1) to ensure Hounsfield number accuracy avoiding systematic HU number errors resulting from cupping (or reverse cupping) artifacts. The cylinders were distributed on six levels each consisting of an inner and an outer circle. The inner circle accommodated a maximum of three cylinders, the outer eight. Since the total number of cylinders scanned was considerably smaller than available positions in the phantom, the cylinders were distributed leaving unfilled positions minimizing beam hardening effects. Scanning was performed at 70, 80, 100, 120 and 140 kVp applying a modified head protocol with 32 times 0.6 mm total collimation, and a medium soft tissue kernel (H40s). The rotation time was set to 1 s and a pitch factor of 0.55 was selected to allow higher effective mAs resulting in reduced image noise compared to clinical protocols. mAs were set to their respective maximum values for 70 kVp (900 mAs) and 80 kVp (1,100 mAs) resulting in a CTDI_vol_ of 31 and 60 mGy, respectively. For 100–140 kV mAs were set to result in a CTDI of 100 mGy (±2 mGy). Slices were reconstructed with 1 mm slice thickness and increment.

**FIGURE 2:**
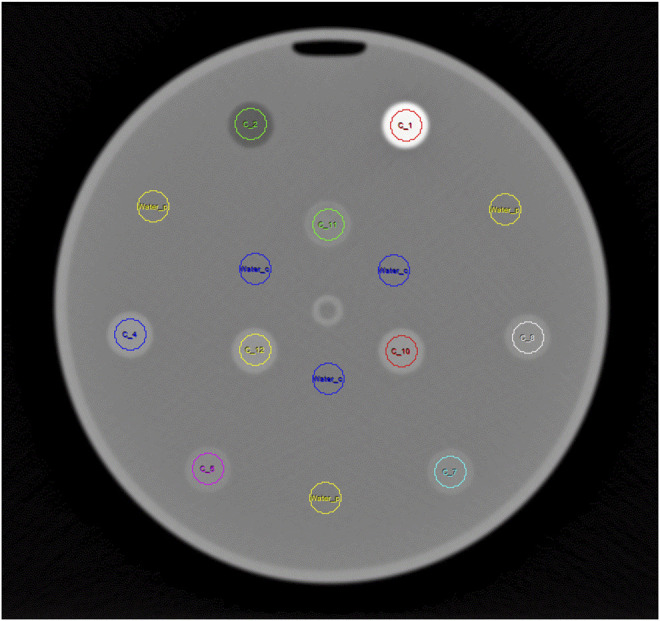
Sample CT slice showing ROIs used for HU measurement in the printed cylinders arranged in two circles. Additional ROIs used to measure HU in the water are shown in blue (inner circle) and yellow (outer circle).

CT images were evaluated with Analyze 12.0, Biomedical Image Resource (Mayo Clinic, Rochester, United States). To avoid HU number inaccuracies from partial volume effects, the outermost slices for each cylinder were excluded from the analysis, and the respective ROIs were smaller (10 mm) compared to the actual diameter of the cylinders of 15 mm (Figure 2). The evaluated height of every 20 mm high cylinder comprised 17 adjacent 1 mm thick slices avoiding top/bottom slices for the same reason. HU values measured for the printed samples were corrected by subtracting the actually measured Hounsfield value in water in the corresponding circle to compensate for eventual cupping and beam hardening not perfectly corrected by the scanner’s beam hardening correction. Therefore, three additional ROIs each were placed in the water filled background at the diameter of the inner, and the outer circle, respectively (Figure 2).

## Results

### Optimization of Printing Parameters for FDM

Printing parameters resulting in optimum print quality, dimensional accuracy and the maximum achievable mass density are summarized in Table 3 for all 25 thermoplastic filaments printed using fused deposition modeling. Five thermoplastic polymer filaments could be printed with a mass density exactly resembling the filament density (± 0.00 g/cm^3^) indicating perfectly solid printouts. These included vinyl, polylactic acid and polycarbonate, as well as filled materials based on PLA/PHA and modified PETG. Eleven materials printed with a deviation of 0.01 g/cm^3^ indicating near optimum achievable density. These materials included all tested PET and modified PET/PETG materials, ABS, ASA and polypropylene. Nine of those exhibited a density 0.01 g/cm^3^ lower than specified filament density, while two (a wood filled PLA, and TPU) exhibited a density 0.01 g/cm^3^ higher than specified.

**TABLE 3:** Optimized printing parameters for the production of phantoms with reproducible x-ray attenuation properties resulting in highest achievable material packing/density.

Material	Nozzle/Bed Temp.[°C]	Printing speed [mm/s]	Flow rate (%)	Max. layer thickness [mm]	Density of printed sample [g/cm^3^]	Filament density [g/cm^3^]	Difference in density
High attenuation filaments							
Vinyl	230/80	40	100	0.15	1.35	1.35	0.00%
PLA/stone	220/60	50	105	0.06	1.64	1.70	−3.53%
PLA/chalk	210/70	60	115	0.06	1.39	1.40	−0.71%
Filled filaments with medium and low attenuation							
PLA-PHA/Glow	210/60	50	110	0.15	1.24	1.24	0.00%
PLA-PBAT bio carbon	220/80	60	115	0.06	1.35	1.15	17.39%
PLA/Al	210/60	50	102	0.06	1.27	n/a^a^	n/a
PLA/wood	200/60	70	115	0.06	1.21	1.20	0.83%
PLA-PHA/Cork	230/60	50	100	0.15	1.21	1.18	2.54%
PETG mod./Carbon	230/60	50	110	0.15	1.19	1.19	0.00%
PLA and PLA/PBAT based filaments							
PLA	210/60	50	110	0.15	1.24	1.24	0.00%
PLA 2	210/60	50	105	0.15	1.23	1.24	−0.81%
PLA/PBAT bio	220/80	60	115	0.06	1.35	1.39	−2.88%
PET and PETG based filaments							
PET	220/75	60	115	0.06	1.33	1.34	−0.75%
PET mod.	260/70	40	105	0.06	1.26	1.27	−0.79%
			110	0.15			
PET mod. 2	240/60	60	115	0.10	1.19	1.20	−0.83%
PETG mod.	220/75	60	115	0.10	1.26	1.27	−0.79%
ABS, ASA and ASA based filaments							
ABS	250/80	60	110	0.06	1.07	1.08	−0.93%
ASA	260/100	50	115	0.06	1.06	1.07	−0.93%
ASA mod.	260/100	50	100	0.15	1.13	1.11	1.80%
PS, PP, PC and Polyamid based filaments							
HIPS	240/100	40	110	0.10	1.02	1.04	−1.92%
PP	240/100	25	100	0.15	0.88	0.89	−1.12%
PP light	240/100	25	115	0.15	0.73	0.75	−2.67
PC	250/90^b^	40	100	0.06	1.20	1.19–1.20	0.00%
Nylon	250/60	45	110	0.15	1.12	1.14	−1.75%
PU based filaments							
TPU	230/70	30	100	0.15	1.16	1.15	0.87%

a Not specified by manufacturer.

b Cooling fan off.

One carbon fiber filled biopolymer exhibited a density 17.39% higher than specified, while the same unfilled base filament had a density 2.88% below its specification. However, according to the manufacturer, the specified density for the carbon fiber filled polymer was based on calculations rather than measurements. A cork filled PLA/PHA filament exceeded the specified density by 2.54%, a modified ASA filament by 1.8%. A polypropylene filament filled with glass microspheres used to lower the density exhibited an even 2.67% too low density, and Nylon and HIPS could only be printed with a density 0.02 g/cm^3^ lower than the specified filament density. A further increase of over-extrusion and decrease of layer thickness did not increase the density of these materials any more, but resulted in the identical print densities.

### X Ray Attenuation and Energy Dependence

**FIGURE 3:**
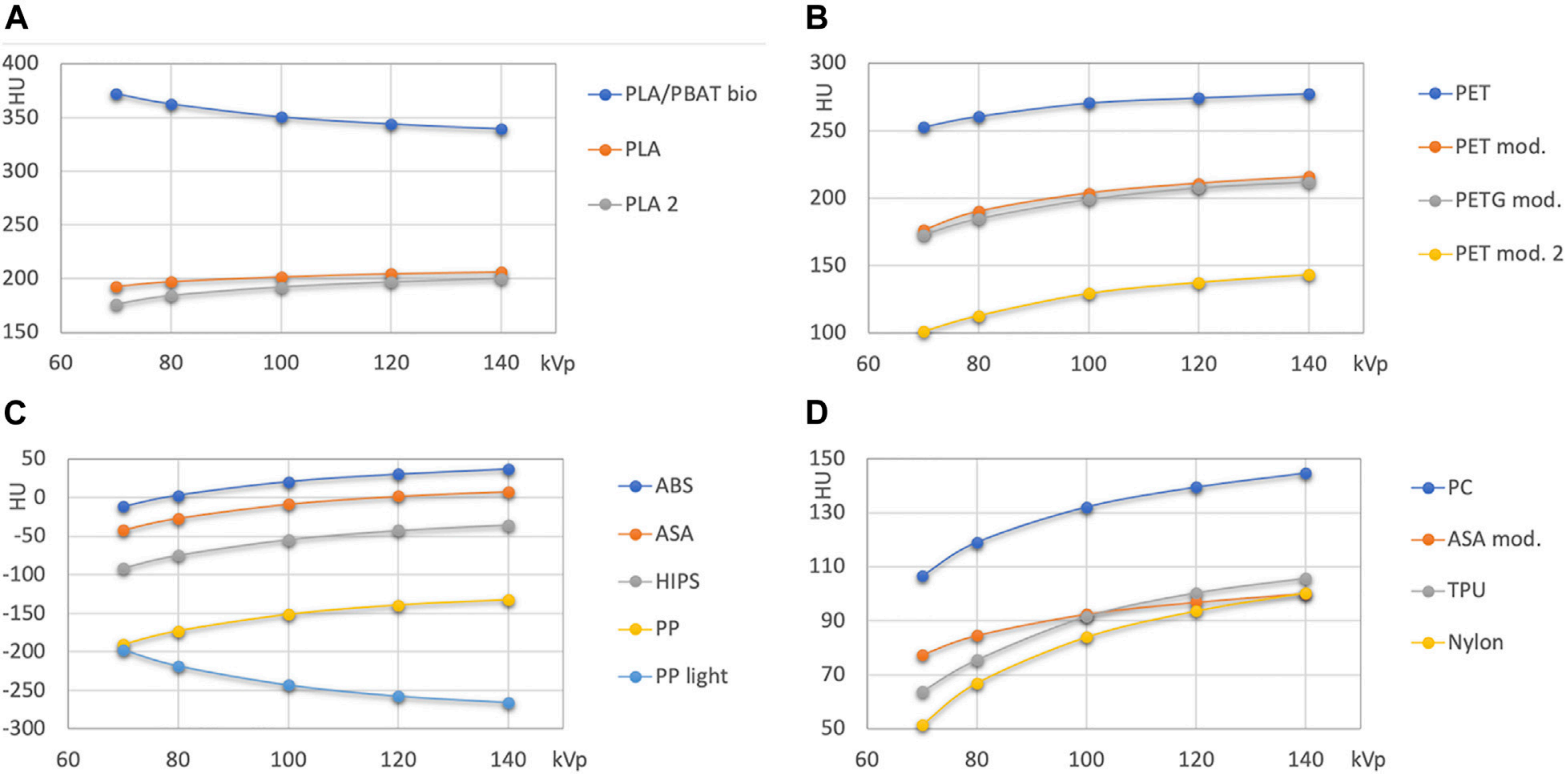
HU values of unfilled base polymers used in FDM printing. **(A)**: PLA and PLA/PBAT; **(B)**: PET and PETG based polymers; **(C,D)**: other polymers with attenuation lower than 50 HU, and with medium attenuation (>50 HU), respectively.

**FIGURE 4:**
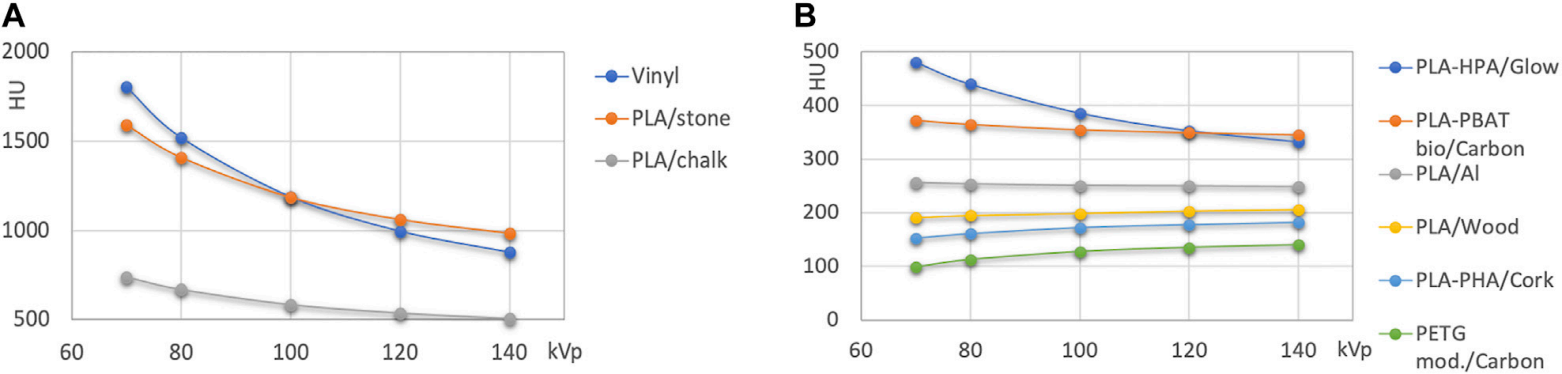
**(A)**: HU values of high attenuation filaments >500 HU. **(B)**: filled polymers with HU values < 500 HU.

Figures 3, 4 show Hounsfield values and their energy dependence for FMM printing materials. In the further discussion, absolute values of HU are stated at 120 kVp, because this tube voltage setting represents the most often used tube potential in CT scanning. The actual exact HU values measured are available in Table 4.

**TABLE 4:** HU values of FDM, polyjet and SLA printing materials in detail.

**PLA and PLA based FDM filaments**				**PET and PETG based filaments**			
**kVp**	**PLA/PBAT bio**	**PLA**	**PLA 2**	**PET**	**PET mod.**	**PETG mod.**	**PET mod. 2**
70	372.1	193.0	176.7	252.8	176.4	172.7	101.0
80	362.7	197.5	184.6	260.6	190.2	184.7	112.7
100	350.7	202.0	192.5	270.5	203.7	199.0	129.3
120	344.1	205.0	197.2	274.4	210.9	207.6	137.5
140	339.6	206.6	200.4	277.6	215.9	212.1	143.3
**ABS, ASA, PS and PP filaments: other polymers with <50 HU**							
	**ABS**	**ASA**	**HIPS**	**PP**	**PP light**		
70	−11.7	−41.4	−90.9	−190.4	−196.9		
80	2.9	−26.5	−74.3	−173.0	−218.0		
100	20.6	−8.2	−54.0	−151.6	−243.2		
120	30.4	2.1	−42.2	−139.6	−257.8		
140	37.0	8.2	−35.0	−132.7	−266.1		
**PC, a modified ASA, TPU and Nylon: other filaments with >50 HU**					**High attenuation filaments**		
	**PC**	**ASA mod.**	**TPU**	**Nylon**	**Vinyl**	**PLA/stone**	**PLA/chalk**
70	106.6	77.6	64.0	51.6	1,804.4	1,592.1	738.6
80	119.1	84.6	75.6	67.0	1,521.2	1,410.2	669.7
100	132.2	92.6	91.6	84.0	1,188.0	1,186.7	584.3
120	139.5	97.0	100.4	93.7	995.8	1,063.1	536.8
140	144.8	100.2	105.9	100.4	877.6	985.8	506.5
**Filled filaments with <500 HU**							
	**PLA-HPA/Glow**	**PLA-PBAT bio/Carbon**	**PLA/Al**	**PLA/Wood**	**PLA-PHA/Cork**	**PETG mod./Carbon**	
70	481.0	372.1	255.7	191.3	153.0	99.3	
80	439.7	364.9	253.4	194.8	161.3	112.9	
100	385.7	354.8	250.6	198.6	172.4	127.7	
120	352.8	349.4	249.6	203.0	177.5	135.3	
140	331.8	345.7	248.3	205.9	181.9	140.2	
**Resins for SLA and Polyjet printing: rigid**							
	**Vero white**	**PCre clear**	**AC translucent**	**Vero blue**	**FL clear**	**Vero clear**	
70	155.9	139.2	117.3	116.5	108.6	103.8	
80	163.6	150.4	129.8	128.3	121.1	117.3	
100	171.3	162.7	142.9	141.5	135.3	133.3	
120	175.7	169.7	150.7	149.0	143.5	142.1	
140	178.3	174.3	155.2	153.5	148.5	147.2	
**Resins for SLA and Polyjet printing: flexible**							
	**PCen flex**	**FL flex**	**Tango**	**FL elastic**			
70	118.9	65.8	56.2	15.8			
80	130.4	77.3	68.1	28.4			
100	142.6	90.7	81.4	43.2			
120	149.7	98.1	89.3	52.0			
140	154.4	102.8	93.8	57.4			

Figure 3 presents the results for nominally unfilled materials (i.e., printing filaments where no filler material such as wood, carbon fiber or mineral powder is specified). In Figure 4 high attenuation filaments potentially mimicking bone tissues, and filled filaments (with declared fillings) are shown.

Natural color and pigmented PLA from two different vendors exhibits similar, but not identical HU values (difference 8 HU at 120 kVp, maximum difference 16 HU at 70 kV; Figure 3A). The attenuation of the transparent PLA1 is slightly higher than the attenuation of PLA2 containing a silver color pigment. The PLA/PBAT based biopolymer exhibits a higher attenuation with approximately 350 HU at 120 kVp.

PET and modified PET/PETG based printouts exhibit a wide range of HU from <140 HU at 120 kVp to >270 HU (Figure 3B).

ABS, ASA, high impact polystyrene (HIPS), and polypropylene (PP) form the group of polymers with the lowest x-ray attenuation. In this group, only ABS exhibits an x-ray attenuation exceeding the one of water at 120 kVp, as it reconstructs with 30 HU. At this energy, ASA mimics water almost perfectly (+2 HU). HIPS (−40) is found close to adipose tissue, and PP is found slightly below −140 HU. The PP light filament exhibits lower HU, with the highest value of approximately −200 HU at 70 kVp and the lowest at the highest energy (<−260). In Figure 3D the remaining printing materials with higher HU values than soft tissues (>90 to 140 HU at 120 kVp) are summarized.

High attenuation filaments serving as potential candidate materials for mimicking hard tissues with different bone mineral content are shown in Figure 4. Besides Vinyl, two different stone/chalk filled PLA based materials exhibited approximately 500 and 1000 HU at 120 kVp. Filled FDM printing polymers (Figure 4B) were found at attenuations from slightly above 100 to approximately 350 HU at 120 kVp.

**FIGURE 5:**
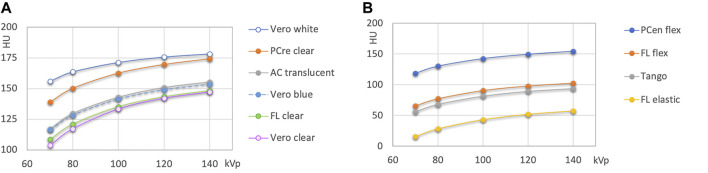
HU values of polyjet and SLA printing resins. **(A)** rigid, and **(B)** flexible resins.

Compared to the wide spectrum of x-ray attenuations found in FDM printing materials, the spectrum of available x-ray attenuations is lower in the resin-based printing technologies (Figure 5). Generally, lower x-ray attenuations can be realized with flexible resins as compared to rigid ones. However, there is an overlap. HU values ranged from slightly over 50 to slightly over 170 at 120 kVp. In the Vero polyjet resins, the clear resin (142 HU at 120 kVp) exhibits the lowest attenuation, and the white one (176 HU) the highest indicating the addition of white mineral pigment, possibly titanium oxide.

For most printing materials irrespective of the printing technology the Hounsfield value increases with increasing beam hardness corresponding to a lower effective atomic number than water and soft tissues except adipose tissue. Regarding unfilled polymers, the only true exemption is Vinyl owned to the higher atomic number of Chlorine. As can be seen in the energy dependence of HU numbers, the PLA/PBAT biopolymer contains a filling with a higher effective atomic number than water, most likely talcum. In PP light, a filament with a reduced mass density of 0.75 as compared to 0.88–0.92 g/cm^3^ for standard polypropylene, embedded air-filled microspheres reduce density. However, these glass microspheres increase the effective atomic number of the material seen in a decrease of HU values with beam hardness.

## Discussion

Available printing materials exhibit a wide range of x-ray attenuations from below -150 to over 1,000 Hounsfield when printed with maximum achievable material density. This range allows the production of radiologic phantoms over a broad gamut of contrasts covering most of the range of attenuations found in patients. However, having established printing parameters resulting in the maximum achievable density, lower attenuations can deliberately and controllably be produced by using infill patterns with reduced filling factor. Many researchers have experimented with varying infill density and patterns to adjust x-ray attenuation properties (Dancewicz et al., 2017) to mimic body tissues. However, this allows a downward adjustment of attenuation, but does not account for the mostly inappropriate energy dependence of most additive manufacturing materials intended to imitate soft tissues like muscle or organ parenchyma. As also stated by Shin et al. (2017) in most polymers tested, HU values increased considerably with increasing tube potential contrary to tissue; in typical soft tissue, Hounsfield numbers only vary minimally with kVp due to their radiological water equivalence, and thus, very similar effective atomic number. The too low effective atomic number (Z_eff_) of most printing materials with attenuation in the soft tissue range or slightly above results in a HU value increase with harder beams. This can be compensated by adding a filler with higher atomic number elements. A good example for this is the PLA/Al filament, e.g., exhibiting an almost constant HU value for all spectra measured. However, since unfilled PLA exhibits already a too high density (1.24 g/cm^3^) and thus linear attenuation coefficient, PLA/Al–despite of the flat energy dependence–cannot serve as a radiological tissue substitute. However, if printed with a lower filling factor the density and attenuation can be reduced in a controllable and reproducible manner. To achieve this goal with a compact phantom material, base polymers with negative HU values like polystyrene or polypropylene (or a mixture of both) would need to be used and higher Z_eff_ materials added (Homolka et al., 2002). A proven choice would be MgO and CaCO_3_. However, such filaments are not available off the shelf.

The achieved densities of the printouts using fused deposition modeling filaments indicated, that most materials can be printed with a density deviation from filament density equal or less than 0.01 g/cm^3^ without quality issues like optically detectable artifacts from over-extrusion. However, print settings have to be optimized for each filament individually and carefully since achieved densities may vary in different batches, between printers and between filament manufacturers (Craft et al., 2018). This tedious process includes printing numerous samples with different settings. To illustrate the procedure followed in this work, it is best described by an example. In the case of high impact polystyrene (HIPS) this procedure involved the following steps: Starting from the parameters optimized for general purpose print jobs resulting in a density of 0.99 g/cm^3^ compared to the filament density specified as 1.04 by the manufacturer, flow rate was increased from 100 to 110% resulting in a density increase to 1.01 g/cm^3^, since flow rate directly influences mass density, and thus can effectively be used to control the latter (Okkalidis, 2018). In the next step, printing with 115% flow rater failed because of over-extrusion artifacts. Decreasing layer thickness instead to 0.1 mm produced a density of 1.02 g/cm^3^ still below specifications. However, decreasing layer thickness further to 0.06 mm did not change density. As a result, 0.1 mm layer thickness, and 110% flow rate were defined as the parameters resulting in the highest achievable density as close to raw filament density as possible, even if the specified filament density could not be reproduced in the printout by 0.02 g/cm^3^ or −1.92%.

Also, different infill patterns and wall thickness settings were tested. To achieve a compact printout, lines or circles can be used as infill pattern. However, generally, using lines resulted in a higher density with all other settings identical in the setup used in this work. The same was found for thin versus thicker outer walls. Therefore, for all printed samples the wall thickness was set to its minimum value, and the infill pattern to lines.

Similar polymers from different manufacturers exhibited different properties. This was seen in the case of the most frequently used and supposedly most easy to print thermoplastic polymer, PLA (polylactic acid). The filament supplied from the printer manufacturer (Ultimaker BV, Utrecht, Netherlands) printed with exactly the density specified and typical for PLA (1.24 g/cm^3^) using 110% flowrate. On the contrary, with the other PLA sample tested these 10% over-extrusion could not be used because of artifacts, and the density specified could not be achieved. This indicates, that filaments from different sources need to be checked on individual basis. However, it needs to be acknowledged that common printing filaments are not specified or optimized for this kind of application.

SLA and polyjet technologies using photocured liquid resins allow for a greater spatial resolution in the prints, but less flexibility in the range of x-ray attenuations. Polyjet technology resins can be mixed during the printing process allowing to print even color gradients. This method allows utilizing the differences in x-ray attenuation of the base materials (the Vero materials in the Stratasys machines, e.g.) to generate phantoms with embedded structures with a selectable radiographic contrast. The highest attenuation was seen in the white material, and a lower attenuation in the transparent. Kiarashi et al. (2015) measured x-ray attenuations of the polyjet printing materials at mammography beam qualities, and found that Vero grey and blue exhibit x-ray attenuation in between white and transparent, and black well below the transparent resin expanding the printable contrast scale.

In the literature various attempts to classify the attenuation of common additive manufacturing materials are available. However, the number of materials tested is more limited than in this work, and some of these need to be read with care. Shin et al. (2017) measured candidate materials for use as analogs for bone, soft tissue, water, and fat from CT scans of filament polymers. The authors scanned a 6 cm radius phantom containing also resolution patterns and samples printed with different infill percentages. 14 printing materials were evaluated at 80 to 140 kVp. However, in the paper no explicit suggestions for candidate materials mimicking bone, soft or adipose tissue, or water, are stated. At 120 kV, the HU value of the materials described range from −55 to 299 HU. Using 100% infill at regular flow rate resulted in potential underfilling (“in several instances, these areas were visibly less dense”), therefore deliberate overfilling was used to result in solid printouts. Due to the overfilling, a resolution pattern intended by design to represent 1.6 line pairs per mm was used to represent the solid material. At 120 kV nylon was measured with 59.1 HU (93.7 HU in this work) indicating 100% filling ratio was actually not reached. The same holds true for ABS, where negative HU values at 120 kVp were reported (ABS red: −49,6, ABS black: −45.2), compared to +30.4 HU in this work. ABS white is reported with slightly positive HU (7.3) most likely due to the white pigment contained. HIPS was measured with −54.7 HU (120 kVp), compared to −42.2 HU in this work, and PLA with 168.5 (red) and 181.1 (clear) compared to 205.0 here. PET measured corresponded to an industrial PETT filament, and exhibited 165.0 (clear) and 177.9 HU (green) which is also very well below the measurements in this work relating to regular PET. However, the chemical composition of the filament used by Shin et al. is not disclosed by the manufacturer.

In Silvestro et al. (2020) also imaging properties of additive manufacturing materials have been determined for potential use in phantoms, focusing on a wide variety of modalities (ultrasound, MRI and CT). The evaluation in CT was limited to the use of automatic kV selection by the scanner, not allowing to derive information on energy dependence. In addition, the interpretation of the results is complicated by not reporting the actual kVp value selected by the scanner. The authors determined the CT numbers of solid ASA and ABS with 16.6 and 57.5 HU, respectively, and Tango and VeroClear with 98.1 and 146.6 HU. In this work, employing defined and appropriate scan conditions by embedding the samples into a water filled cylinder, plain ASA and ABS (without filling materials and color pigments) were measured with at 2.1 and 30.4 HU at 120 kV, respectively, and much lower values at lower kVp, and higher values for harder spectra. Tango and Vero clear were found in this work at 89.3 and 142.1 HU (120 kV). However, the phantom used by Silvestro et al. (2020) consisted of 4.5 × 2.6 × 0.65 cm polymer samples evaluated, embedded into a poured silicone block 75 mm high. The form, size, and material of the embedding will not allow the CT’s built-in beam hardening correction to derive correct HU numbers of the samples. Especially silicone embedding should be avoided because of the too high atomic number of silicon resulting in issues with the beam hardening correction aiming at avoiding cupping and reverse cupping in patients and radiographically water equivalent materials only. HU measured this way will most likely not reflect those measured in more appropriate scan condition as used in this work. This discrepancy outlines the importance of using appropriate scan conditions including reporting technical scan parameters like kVp if correct and reproducible HU numbers shall be reported.

Bibb et al. (2011) measured CT values of 14 solid additive manufacturing material samples of 40 × 20 × 10 mm embedded in a low density foam block (“free in air”), and attached to a head phantom at 120 kV using a bone reconstruction kernel. However, no care was taken to ensure a complete filling of the FDM printed samples, and the CT image provided in the figure clearly exhibits a linear inner structure of the samples, indicating inhomogeneities due to underfilling. The authors state that “some samples are not homogeneous and their density varies considerably across their sections”, indicating that CT numbers were strongly influenced by the printing process and might not be attributed to the material attenuation properties only, as desired. However, this example clearly shows the necessity to optimize the printing parameters (mostly, layer height and deliberate over-extrusion in FDM) very carefully for every material and individual printer before printing phantoms to avoid underfilling or inhomogeneous densities. Also, scanning conditions influence the CT numbers mostly due to the effect of the beam hardening correction. In Bibb et al. (2011) up to 128 HU difference (average 28 HU) is reported for the same sample scanned either on the head phantom, or in the foam block. This difference, again, emphasizes the importance of well-defined scanning conditions, as is best demonstrated by CT quality control or electron density calibration phantoms used in radiotherapy planning (Constantinou et al., 1992; Nakao et al., 2020), where the calibration cylinders are embedded into a water equivalent material in a cylindrical phantom of appropriate diameter.

Other studies aimed at suggesting commonly available additive manufacturing materials explicitly for mimicking tissue in radiographic phantoms. Kairn et al. (2020) concluded, that PLA filaments with a Calcium based filling, like StoneFil (Formfutura BV, Nijmegen, Netherlands) represent an appropriate surrogate for hard bone at kV and MV energies which is in accordance with this study. Ivanov et al. (2018) studied the suitability of common low density 3D printing materials for the fabrication of breast phantoms. Their conclusion was, that most SLA resins, as well as Nylon and PET-G exhibit attenuation characteristics close to glandular tissue, while ABS more closely mimics adipose tissue at mammographic energies, and thus, represent possible material candidates for 3D printed phantoms.

Printing different materials in one phantom is limited to few printing technologies, like, e.g., polyjet printing. Some FDM printers using a dual head extrusion system are limited to printing two materials simultaneously. However, phantoms parts made of different materials, and even made with different technologies possibly combining the high spatial resolution from photocured resins with the wide range of x-ray attenuation found in filled FDM filaments, can be printed separately and assembled. Another possibility is using castable materials filled in voids of the printed base phantoms, as has been done in (Hatamikia et al., 2020) e.g., where soft tissue was printed and bone mimicking material then cast in the voids spared in the printing process using bone mineral and polypropylene powder mixed into epoxy resin. In (Cockmartin et al., 2017) anthropomorphic breast lesions were printed, and assembled into a phantom filled with PMMA speres and water simulating the anatomical background.

## Conclusion

When determining the radiation attenuation properties of additive manufacturing materials (and, materials in general), printing parameters and scan conditions need to be selected carefully and appropriately, what has not always been the case in published studies. For FDM printing, it is indispensable to assure the maximum achievable filling ratio is achieved prior to printing the test samples.

The energy dependence of low and medium attenuation additive manufacturing polymers is mostly unfavorable due to the low effective atomic number. While the linear attenuation coefficient, of course, decreases with photon energy, it decreases less strongly than in water, resulting in a relative increase of HU values with harder beams. This makes it difficult to print materials with linear attenuation coefficients similar to muscle tissue or organ parenchyma over an extended energy range. However, spectrum and energy independent attenuation relative to water can be realized by adding filler materials with a higher atomic number. However, in readily available industrial and consumer grade printing materials the choice is limited. For high attenuation tissues like bone tissues, calcium filled polymers provide both, favorable energy dependence, and attenuation values.

The measurements performed in this study can be used to design phantoms with a wide range of x-ray contrasts. Using the information on the energy dependence from 70 to 140 kVp, materials with similar energy dependence can be selected to allow (almost) energy independent contrasts. If the energy dependence of the intended phantom contrast is known, the most appropriate materials may be selected and the absolute value of the linear attenuation adjusted downwards by using under extrusion and adjusted infill ratios. The data provided on the energy dependence can also be used to correct contrast or contrast to noise ratios measured in phantoms made from a wide selection of additive manufacturing materials to simulate anatomical contrasts from tissues with known, but different energy dependence. These include contrasts between different soft tissues (organ parenchyma, muscle, adipose tissue to name some of the most important), lung tissue, and bone of various densities.

## Data Availability

The original contributions presented in the study are included in the article/Supplementary Material, further inquiries can be directed to the corresponding author.
